# Coinfection with HIV-1 Alleviates Iron Accumulation in Patients with Chronic Hepatitis C Virus Infection

**DOI:** 10.1371/journal.pone.0098039

**Published:** 2014-06-13

**Authors:** Yuan Liu, Quanjun Lv, Jian Gao, Lu Long, Zhaojun Duan, Hua Liang, Tao Shen, Fengmin Lu

**Affiliations:** 1 Department of Microbiology & Infectious Disease Center, Peking University Health Science Center, Beijing, China; 2 Department of Nutrition and Food Hygiene, College of Public Health, Zhengzhou University, Henan, China; 3 State Key Laboratory for Infectious Disease Prevention and Control, National Center for AIDS/STD Control and Prevention, Chinese Center for Disease Control and Prevention, Collaborative Innovation Center for Diagnosis and Treatment of Infectious Diseases, Beijing, China; Lady Davis Institute for Medical Research/McGill University, Canada

## Abstract

Most chronically-infected hepatitis C virus (HCV) patients have increased levels of iron in the liver. Iron overload reduces sustained responses to antiviral therapy, leading to more rapid progression to liver cirrhosis and the development of hepatocellular carcinoma. However, it is still unclear how HIV-1 infection affects iron status in patients chronically infected with HCV. The present study recruited 227 patients from a village in central China. These patients were either monoinfected with HCV (n = 129) or coinfected with HCV/HIV-1 (n = 98). Healthy controls (n = 84) were also recruited from the same village. Indicators of iron status, such as serum levels of iron, ferritin, and transferrin, total iron-binding capacity (TIBC), transferrin saturation (Tfs), and hepcidin, were analyzed and compared across the three groups. The results showed that serum levels of iron (p = 0.001) and ferritin (p = 0.009) and the Tfs (p = 0.002) were significantly higher in HCV-monoinfected patients than in the healthy controls; however, there were no differences in iron levels and Tfs between HCV/HIV-1 coinfected patients and healthy controls. Additionally, although serum hepcidin levels in HCV-monoinfected and HCV/HIV-1-coinfected patients were lower (p<0.001) than those in health controls, the levels in coinfected patients were higher (p = 0.025) than those in HCV-monoinfected patients. Serum iron and ferritin levels in HCV-monoinfected patients were positively correlated with serum ALT/AST. Serum transferrin levels were negatively correlated with ALT/AST levels. The levels of iron in the serum of coinfected patients with a CD4+T-cell count <500/µl were lower than those in patients with a CD4+T-cell count ≥500/µl, whereas serum hepcidin levels showed the opposite trend. Taken together, these results suggest that coinfection with HIV-1 alleviates iron accumulation caused by chronic HCV infection. Our study indicated that determining the status of serum iron and other iron-associated parameters will be helpful to understand the complexity of alternations in iron distribution in HCV/HIV-1-coinfected patients.

## Introduction

Hepatitis C virus (HCV) infection is a major cause of chronic liver disease. Even though approximately 170 million people worldwide are infected with HCV [1], it is still unclear how HCV infection causes liver injury and carcinoma. The cytotoxic effects of virus core proteins, chronic inflammation, and recurrent/prolonged immune responses have all been suggested as cofactors responsible for liver damage [2], [3]. A central hypothesis for disease pathogenesis is associated with excess iron load, which may be driven by chronic inflammation [4]. Many experimental and clinical studies [2], [5]–[7] suggest that chronic iron deposition promotes the progression of liver damage and increases the risk of fibrosis, cirrhosis, and hepatocellular carcinoma (HCC) in chronic hepatitis C (CHC) patients [6], [7]. Accumulating evidence [8]–[10] suggests that excess iron catalyzes the formation of highly reactive free radicals and hydroxyl radicals, which can damage lipids, proteins and DNA. Liver satellite cells are susceptible to oxidative injury, and are easily transformed into collagen-producing cells that contribute to the development of fibrosis [11]. Furthermore, some studies [12], [13] suggest that excess iron in the liver may have an adverse effect on a patient's response to antiviral therapy [13].

Many hypotheses [14]–[16] have been put forward to explain the accumulation of iron in the liver of patients with CHC, including hemochromatosis mutations, loss of iron from damaged hepatocytes, and HCV-induced disturbance of iron homeostasis. The mechanism(s) underlying iron accumulation in HCV-infected livers is unclear; however, the recent discovery of hepcidin, a peptide hormone that regulates iron hemostasis, may provide some clues. Alterations in iron distribution are common in infectious diseases and many of these alterations may be attributable to actions of the iron-regulatory hormone hepcidin. Hepcidin degrades the sole cellular iron exporter ferroportin leading to reduced iron absorption in the intestine and iron retention in monocytes, macrophages and spleen [17], [18].Several studies [19], [20] report increased or decreased iron levels in the serum of HIV patients (depending on the stage of the disease). Also, HIV-associated anemia is reported common among HIV-infected patients in Indonesia and strongly related to mortality. High ferritin with low soluble transferrin receptor (sTfR) levels suggest that iron redistribution and low erythropoietic activity, rather than iron deficiency, contribute to anemia [21]. Impact of coinfection with HCV may further complicate the assessment of iron status [12]. In addition, a clinical study [19] reported macrophage iron loading in HIV-infected patients. Since some fundamental cellular processes, including DNA synthesis and the generation of ATP, also require iron, HIV replication may suppress iron transport from macrophages into the serum, resulting in iron accumulation in these cells [20], [22], [23].

Because HCV and HIV share routes of transmission in China, up to 90% of HIV-1-infected former blood donors (FBDs) are positive for anti-HCV antibodies. Although HCV infection is associated with dysfunctional iron metabolism and hematological fluctuation, the effects of coinfection on the iron status of HCV-infected patients are still unknown. Here, we examined whether coinfection with HIV-1 affects iron levels in HCV-infected patients.

## Methods

### Patients and controls

Two hundred and twenty-seven patients aged between 30 and 55 years were recruited from a village of Henan province in 2009. Of these, 129 (56.8%) were infected with HCV (monoinfected) and 98 (43.2%) were infected with both HCV and HIV-1 (coinfected). More than 90% of patients were FBDs; the remainder comprised their parents, spouses, or children. All were negative for hepatitis B virus surface antigen. HCV-monoinfected patients had detectable serum HCV-RNA, were positive for anti-HCV antibodies, and showed no anti-HIV immune responses. HCV/HIV-coinfected patients had detectable HCV-RNA, and were positive for anti-HCV and anti-HIV antibodies. All of the HIV-positive patients had received highly active antiretroviral therapy, either regularly or intermittently. The healthy control group comprised 84 patients from the same village. These patients were negative for HBsAg, negative for anti-HCV and anti-HIV antibodies, and had ALT and AST levels <40 IU/L. None of the patients had taken iron supplements during the 2 months prior to enrollment, and none of the female patients was pregnant.

A fasted venous blood sample was collected from each participant. Serum and EDTA anti-coagulated plasma were stored at −70°C until use. Routine blood tests, clinical biomedical tests, and CD4+/CD8+T-cell counts were performed by the local CDC. The study was approved by the institutional review board of Peking University Health Science Center, and all patients provided written informed consent.

### HCV status

Serum samples were tested for the presence of anti-HCV antibodies using the Abbott Architect anti-HCV chemiluminescent microparticle immunoassay (Abbott GmbH & Co.KG, Wiesbaden, Germany). HCV-RNA levels were measured using the Realtime HCV Amplification Kit (Abbott Molecular Inc., Des Plaines, IL). The detection limit was 20 IU/ml.

### HIV status

HIV-1 screening was performed using an HIV-1/-2 antibody enzyme immunoassay (GBI Biotech Co., Ltd., Beijing, China). Positive samples were confirmed in an HIV Blot 2.2 Western blot assay (Genelabs Diagnostics, Singapore). HIV-RNA levels were measure using Cobas Amplicor HIV-1 Monitor Test version 1.5 assay (Roche Molecular Systems Inc., Durham, NC). The detection limit was 50 copies/ml.

### Determination of iron-related parameters

Serum hepcidin concentrations were measured in a competitive enzyme-linked immunosorbent assay (ELISA) (Bachem S-1328). Briefly, 96-well plates were coated overnight with a goat anti-rabbit IgG (Fc) antibody at 4°C, blocked with bovine serum albumin for 2 h, and then incubated with a rabbit anti-human hepcidin antibody for 2 h. The study samples, and a biotinylated hepcidin-25 (25 amino acids) calibrator peptide were added to the wells and incubated overnight at 4°C. The plates were then incubated with conjugate for 1 h. Finally, substrate solution was added for 15 min. The color reaction was stopped and the absorbance measured at 492 nm in an automated ELISA reader.

Serum iron/TIBC was measured using a ferrozine-based colorimetric method in a HITACHI LABOSPECT008 automatic biochemical analyzer. Serum ferritin and transferrin were measured in the Architect Ferritin assay (Abbott) according to the manufacturer's instructions. Tfs% was calculated according to the following formula: Tfs% = (serum iron concentration/TIBC)×100.

### Statistical analysis

Quantitative data were expressed as numbers and percentages, and continuous variables as the median and inter-quartile range (IQR). Differences between two groups were determined using Student's *t* test. Differences between the three groups were compared using one-way analysis of variance. The Kruskal–Wallis test was used when data were not normally distributed. Categorical variables were compared using the Chi-squared test or Fisher's exact test, as appropriate. Pearson's or Spearman's tests were used for correlation analysis. A two-sided *P*-value of <0.05 was considered statistically significant. All statistical analyses were performed using the SPSS (17.0) software (Chicago, IL, USA).

## Results

### Patient characteristics

Patients and controls were compared in terms of age, sex, Body Mass Index (BMI), serum HCV viral load, HCV genotype, smoking and alcohol consumption, and relevant liver indicators (ALT, AST, albumin, and total bilirubin). There were no differences among the three groups in terms of age, sex, BMI, serum HCV viral load, HCV genotype, or smoking and alcohol consumption. Compared with the healthy controls, HCV-positive patients had higher ALT and AST levels, which are a part of diagnostic indicators of intrahepatic inflammation or hepatocellular injury. HCV-infected patients had lower serum albumin levels than the healthy controls. The clinical and biochemical characteristics of the patients and the controls are shown in Table S1.

### Comparison of hematological parameters among the three groups

The hematological parameters for the HCV-monoinfected and HCV/HIV-1-coinfected patients and the healthy controls are summarized in Table 1. The red blood cell count (RBC), hemoglobin (Hb) level, hematocrit (HCT) level, mean corpuscular volume (MCV), mean corpuscular hemoglobin (MCH), and mean corpuscular hemoglobin concentration (MCHC) were higher in HCV-monoinfected patients than in healthy controls; however, the MCV, MCH, and MCHC were significantly lower in HCV-monoinfected patients than in HCV/HIV-1-coinfected patients, whereas RBC, Hb, and HCT were higher in HCV-monoinfected patients than in coinfected patients. HCV-monoinfected and HCV/HIV-1-coinfected patients had lower platelet counts than healthy controls.

**Table 1:** Hematological parameters for the three groups.

Variables	HCV monoinfection (n = 129)	*P^a^*	HCV/HIV-1 coinfection (n = 98)	*P^b^*	Healthy controls (n = 84)	*P^c^*
RBC (10^12^/L)		*0.003*		***<0.001***		***<0.001***
* Median*	5.23		4.32		4.83	
* IQR*	4.77–5.67		3.72–4.91		4.32–5.37	
WBC (10^9^/L)		*0.86*		***0.02***		***0.03***
* Median*	6.30		5.55		6.21	
* IQR*	5.10–7.45		4.43–7.10		5.60–7.68	
PLT (10^12^/L)		***<0.001***		***<0.001***		***<0.001***
* Median*	222.0		151.5		242.0	
* IQR*	166.5–251.5		115.3–194.8		179.8–339.0	
Hb (g/L)		***<0.001***		*0.09*		***<0.001***
* Median*	142.1		121.4		130.8	
* IQR*	128.2–154.5		113.2–136.3		114.2–145.0	
HCT (%L/L)		***<0.001***		*0.57*		***<0.001***
* Median*	48.9		43.4		44.4	
* IQR*	44.2–53.1		39.5–50.7		39.3–50.3	
MCV (fL)		***0.008***		***<0.001***		***<0.001***
* Median*	93.70		102.5		91.65	
* IQR*	91.30–97.70		93.71–112.3		88.53–94.68	
MCH (pg)		***0.006***		***<0.001***		***<0.001***
* Median*	27.60		30.10		26.55	
* IQR*	26.33–28.70		27.2–34.6		24.92–27.98	
MCHC (g/L)		***0.03***		***0.004***		*0.55*
* Median*	291.3		294.7		287.5	
* IQR*	282.5–300.4		286.2–301.7		271.7–299.8	

IQR: inter-quartile range; Hb: hemoglobin; HCT: hematocrit; RBC: red blood cell; MCV: mean corpuscular volume; MCH: mean corpuscular hemoglobin; MCHC: mean corpuscular hemoglobin concentration. *P* ^a^ refers to the comparison between HCV-monoinfected patients and healthy controls. *P* ^b^ refers to the comparison between HCV/HIV-coinfected patients and healthy controls. *P* ^c^ refers to the comparison between HCV-monoinfected patients and HCV/HIV-coinfected patients.

### Iron status in patients and controls

As shown in Table 2, the median level of serum iron in HCV-monoinfected patients was 18.64 µmol/l and the Tfs was 34.25%; both values were significantly higher than those in HCV/HIV-1-coinfected patients (p = 0.007 for iron and p = 0.004 for Tfs) or healthy controls (p = 0.001 for iron and p = 0.002 for Tfs). However, there were no differences in these values between HCV/HIV-1-coinfected patients and healthy controls. HCV-monoinfected (p = 0.009) and HCV/HIV-1-coinfected (p = 0.003) patients had higher serum ferritin levels than healthy controls.

**Table 2:** Iron-associated parameters measured in HCV and/or HIV seropositive patients and healthy controls.

Variables	HCV monoinfection (n = 129)	*P* ^a^	HCV/HIV-1 coinfection (n = 98)	*P* ^b^	Healthy controls (n = 84)	*P* ^c^
Serum iron (µmol/L)		***0.001***		0.186		***0.007***
* Median*	18.64		15.11		14.91	
* IQR*	14.50–24.69		11.45–20.41		11.5–18.96	
Serum ferritin (µg/L)		***0.009***		***0.003***		*0.11*
* Median*	110.6		120.2		85.4	
* IQR*	81.7–140.3		81.9–144.3		69.3–105.5	
Serum hepcidin (ng/mL)		***<0.001***		***<0.001***		***0.025***
* Median*	56.42		61.90		86.75	
* IQR*	45.60–67.47		49.52–70.21		78.95–92.76	
Serum transferrin (g/L)		***0.021***		***<0.001***		*0.31*
* Median*	2.22		2.18		2.37	
* IQR*	2.03–2.48		1.99–2.38		2.37–2.79	
TIBC (µmol/L)		***0.009***		***<0.001***		*0.13*
* Median*	55.69		54.83		57.22	
* IQR*	50.75–61.15		49.41–59.69		52.34–65.51	
Tfs (%)		***0.002***		0.110		***0.004***
* Median*	34.25		29.38		27.42	
* IQR*	25.20–41.03		20.35–38.88		19.64–37.59	

Data are expressed as the median and inter-quartile range (IQR).

TIBC: total iron-binding capacity; Tfs: transferrin saturation. *P* ^a^ refers to the comparison between HCV-monoinfected patients and healthy controls. *P* ^b^ refers to the comparison between HCV/HIV-coinfected patients and healthy controls. *P* ^c^ refers to the comparison between HCV-monoinfected patients and HCV/HIV-coinfected patients.

Conversely, serum transferrin levels and TIBC were significantly lower in HCV-monoinfected (p = 0.021 for transferrin and p<0.009 for TIBC) and HCV/HIV-1-coinfected (p<0.001 for transferrin and p<0.001 for TIBC) patients than in the healthy controls. The hepcidin concentration in the serum of HCV-monoinfected patients was 56.42 ng/ml, whereas that in the serum of HCV/HIV-1-coinfected patients was 61.90 ng/ml. Both values were significantly lower than that in healthy controls (86.75 ng/ml) (p<0.001). In addition, the serum hepcidin concentration in HCV/HIV-1-coinfected patients was higher than that in HCV-monoinfected patients(p = 0.025).

### Correlation between iron status and liver enzyme levels and CD4+T-cell counts

**Figure 1:**
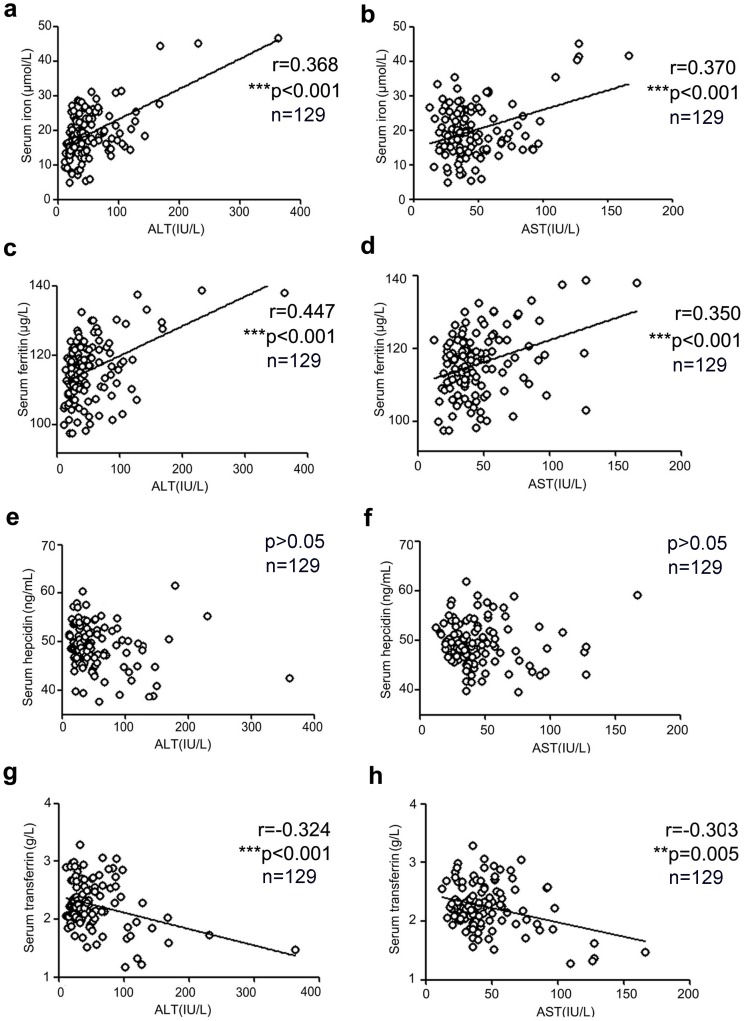
Correlation between serum iron-related parametersand serum ALT (left panel) and AST levels (right panel) in chronically HCV-infected patients. Pearson's or Spearman's tests were used for correlation analysis. A two-sided *P*<0.05 was considered statistically significant. (*** indicates P values <0.001.).

We also analyzed the correlation between of serum iron, ferritin, hepcidin, and transferrin levels and those of ALT and AST in HCV-monoinfected patients (Fig. 1). The serum iron and ferritin levels were positively correlated with serum ALT levels in HCV-monoinfected patients (serum iron: r = 0.368, p<0.001; ferritin: r = 0.447, p<0.001). A significant correlation was also observed for AST (serum iron: r = 0.370, p<0.001; ferritin: r = 0.350, p<0.001). Conversely, serum transferrin levels were negatively correlated with serum ALT and AST levels (r = −0.324, p<0.001 for ALT and r = −0.303, p = 0.005 for AST) in these patients. There was no correlation between hepcidin levels and ALT/AST levels (Figure 1a–h). There was no correlation between serum iron, ferritin, and transferrin levels and AST/ALT levels in HCV/HIV-coinfected patients and healthy controls (data not shown). None of these parameters correlated with peripheral CD4+T-cell counts in HCV/HIV-1-coinfected patients (data not shown).

### Comparison of iron-associated indicators in HCV/HIV-1-coinfected patients with different immune status

**Figure 2:**
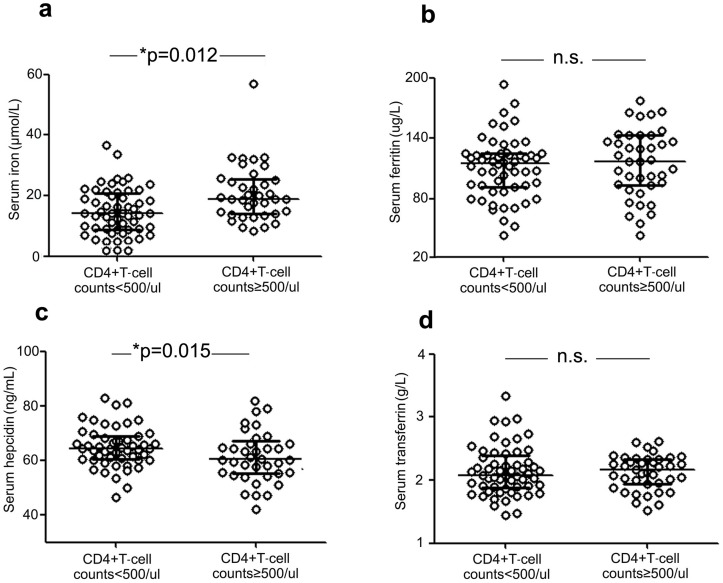
Comparison of iron-associated parameters in HCV/HIV-coinfected patients according to immune status. Serum concentrations of iron (a), ferritin (b), hepcidin (c), and transferrin (d) were measured in HCV/HIV-coinfected patients and then compared with CD4+T-cell counts (<500/µl *vs.* ≥500/µl). Data are expressed as the median and inter-quartile range. Differences between two groups were determined using Student's *t* test and a two-sided *P*<0.05 was considered statistically significant. (Asterisks indicate P values <0.05.).

When we stratified HCV/HIV-1-coinfected patients according to immune status (CD4+T-cell counts <500/µl and CD4+T-cell counts ≥500/µl), we found that immunocompromised patients (CD4+T-cell counts <500/µl) had significantly lower levels of serum iron (p = 0.012) than immunocompetent patients (CD4+T-cell counts ≥500/µl). By contrast, serum hepcidin levels were higher in patients with CD4+T-cell counts <500/µl (p = 0.015). However, there were no differences in serum ferritin and transferrin levels between the two groups (Figure 2). Also, no correlations between HIV viral loads and iron-associated indicators were identified in our study (data not shown).

## Discussion

The liver is the main organ responsible for iron homeostasis, and the status of the liver is closely related to the distribution of iron within the body. Some iron-associated proteins, such as hepcidin and transferrin, are primarily synthesized by hepatocytes [24]. Pathological iron overload is observed in >50% of chronically-infected HCV patients. This is important because increased hepatic iron storage is an independent risk factor for the development of HCC [9], [25].

Here, we found that serum iron and ferritin levels were higher in HCV-monoinfected patients than in healthy controls (Table 2). Iron is stored in the liver in the form of ferritin. When the liver is damaged by viral infection, ferritin is released from the damaged hepatic cells and actively secreted by macrophages during inflammation as well [26]. This leads to higher serum ferritin concentrations in HCV-infected patients. We also found that HCV-infected patients had lower serum concentrations of hepcidin and transferrin. Hepcidin is a 25-amino acid peptide hormone primarily synthesized by hepatocytes, which regulates iron homeostasis by controlling iron absorption in the gut, iron release from macrophages, and the recycling of iron through erythrophagocytosis [27]. In agreement with the results presented herein, several studies report low hepcidin levels in HCV-infected patients [28]. Hepcidin binds to ferroportin, a membrane iron exporter expressed at high levels on enterocytes and macrophages. This results in ferroportin internalization and degradation, and a subsequently reduction in iron levels in the plasma. Recent studies suggest that reduced serum hepcidin levels in patients with chronic hepatitis C are associated with higher Tfs and increased serum iron accumulation. HCV infection may directly modulate hepcidin expression, and that higher Tfs and increased iron accumulation result in resistance to hepcidin [12]. Transferrin mainly functions to regulate iron transport and to prevent excessive iron deposition. Because transferrin is synthesized in the liver, impaired liver function caused by HCV infection results in lower transferrin levels.

Here, our main focus was the effect of HCV/HIV-1 coinfection on iron status. We found that coinfected patients had significantly lower levels of serum iron and a lower Tfs than HCV-monoinfected patients; however, there were no differences between HCV/HIV-1-coinfected patients and healthy individuals. Even though the levels of serum hepcidin were lower in HCV-monoinfected and HCV/HIV-1-coinfected patients than in healthy individuals, the levels were higher in HCV/HIV-1-coinfected patients than in HCV-monoinfected patients. HIV infects CD4+ helper T-cells, macrophages, and dendritic cells [29], all of which play vital roles in the immune response. Macrophages also play an important role in iron homeostasis by recycling around 30 mg of iron per day, which is 20–30-fold greater than the amount absorbed from food [8]. If macrophages are compromised by HIV, much of the iron may accumulate within the cells and is not released into the serum, resulting in reduced serum iron levels in infected patients. Also, Hepcidin may directly suppress the generation of erythrocyte during inflammation [30]. This is consistent with our data showing that the RBC was lower in coinfected patients than in monoinfected patients. The reason for this reduction in the RBC is complicated, and may involve the drugs taken by HIV-infected patients [31], [32]. For example, it is known that for infants, anemia prevalence and serum ferritin level decrease after HAART without routine iron supplementation [33]. All of HIV-infected patients in our study had received zidovudine-containing HAART treatment, either regularly or intermittently. The decreased iron load in HIV-infected patients may be caused by both of HIV *per se* and HAART therapy.

We also examined the correlation between ALT/AST levels and serum iron, ferritin, hepcidin, and transferrin levels. As expected, serum iron and ferritin levels were positively correlated with ALT/AST levels and negatively correlated with transferrin levels in HCV-monoinfected patients. No similar correlations were found in HCV/HIV-1-coinfected patients. Although there was no correlation between CD4+T-cell counts and any of the iron-related parameters, we did find that serum iron and hepcidin levels differed in patients according to immune status. Immunocompromised patients (CD4+T-cell counts <500/µl) had significantly higher levels of serum hepcidin than immunocompetent patients (CD4+T-cell counts ≥500/µl). A study by Xu et al [34] shows that high concentrations of hepcidin induce the transcription of HIV-1 proviral DNA. It may be that coinfection resulted in the viruses cancelling each other out in terms of their effects on iron metabolism, leading to reduced iron accumulation in HCV/HIV-coinfected patients.

Despite the belief that HCV infection can be cured by consistent optimization of therapeutic regimens containing PEG-IFN/Ribavirin, a certain proportion of chronically-infected patients do not respond to treatment [9]. This may be due to different genotypes and single-nucleotide polymorphisms (SNPs) in the IL28B genes; however, dysregulated iron homeostasis may be another reason. Evidence suggests that excess iron exerts toxic and fibrogenic effects via pathways involving the production of oxyradicals and oxidative stress; indeed, several studies show that removing excess iron through therapeutic phlebotomy reduces the severity of hepatic inflammation associated with chronic HCV infection [2]. Accordingly, determining the status of serum iron and other iron-associated parameters, will be helpful to understand the complexity of alternations in iron distribution in HCV-infected patients, particularly those coinfected with HIV-1.

## Limitation

Our study has some limitations. Our cohort lack a manageable number of HIV monoinfected patients. Since HCV is more efficiently transmitted by blood-borne routes than HIV [20], more than 90% of HIV-positive patients in our cohort infected HCV simultaneously (positive anti-HCV at least). Only a small number of the HIV-monoinfected patients were found in the same village, which is no enough to perform convincing statistical analysis. Additionally, because the iron status is influenced by surroundings, diet and nutriture, the other cohort of HIV infection served as a reference may not be the most appropriate selection. In addition, due to no liver biopsy samples were available, liver iron quantification or In situ expression of hepcidin protein have not been performed in our study.

## Supporting Information

Table S1 Clinical and biochemical characteristics of the HCV and/or HIV-1 seropositive patients and the healthy donors.
(DOC)
