# Supplementary material for: Coinfection with HIV-1 Alleviates Iron Accumulation in Patients with Chronic Hepatitis C Virus Infection
Source: PLoS One. 2014 Jun 13;9(6):e98039. doi: 10.1371/journal.pone.0098039 (PMC4057081; doi:10.1371/journal.pone.0098039)
Supplement: Table S1 — Clinical and biochemical characteristics of the HCV and/or HIV-1 seropositive patients and the healthy donors. (DOC) [file pone.0098039.s001.doc]

**Supplementary Table 1. Clinical and biochemical characteristics of the HCV and/or HIV-1 seropositive patients and the healthy donors**

| **Variables** | **HCV monoinfection**  **(n=129)** | ***P^a^*** | **HCV/HIV-1 coinfection**  **(n=98)** | ***P^b^*** | **Healthy controls**  **(n=84)** | ***P^c^*** |
| --- | --- | --- | --- | --- | --- | --- |
| Age, years | 44 (41–52) | *0.23* | 44 (40–53) | *0.37* | 45 (38–52) | *0.34* |
| Sex, n (%) |  | *0.65* |  | *0.71* |  | *0.53* |
| *Female* | 61 (47) |  | 53 (54) |  | 46 (55) |  |
| *Male* | 68 (53) |  | 45 (46) |  | 38 (45) |  |
| BMI | 23.38 (21.15–25.53) | *0.96* | 22.12 (20.60–24.39) | *0.15* | 23.01 (21.13–26.23) | *0.11* |
| Anti-HIV antibodies | Negative |  | Positive |  | Negative |  |
| Anti-HCV antibodies | Positive |  | Positive |  | Negative |  |
| HIV-RNA, log_10_ copies/mL | Negative |  | 4.21 (3.04–5.27) |  | Negative |  |
| HCV-RNA, log_10_ IU/mL | 6.01 (5.43–6.35) |  | 6.53 (5.87–6.79) |  | N.A. | *0.73* |
| HCV genotype |  |  |  |  | N.A. | *0.09* |
| *1b, n%* | 40, 31.0% |  | 41, 42.7% |  |  |  |
| *2a, n%* | 88, 69.0% |  | 55, 57.3% |  |  |  |
| *Others, n%* | none |  | none |  |  |  |
| CD4+T counts, cells/μL | 812 (586–1046) | *0.16* | 409 (292–571) | ***<0.001*** | 879 (656–1029) | ***<0.001*** |
| ALT (n, %) |  | ***<0.001*** |  | ***<0.001*** |  | *0.79* |
| ALT>40 (IU/mL) | 59 (45.7%) |  | 42 (43.8%) |  | 8 (9.5%) |  |
| ALT≤40 (IU/mL) | 70 (54.3%) |  | 54 (56.3%) |  | 76 (90.5%) |  |
| AST (n, %) |  | ***<0.001*** |  | ***<0.001*** |  | *0.93* |
| AST>40 (IU/mL) | 61 (47.3%) |  | 53 (55.2%) |  | 4 (4.8%) |  |
| AST≤40 (IU/mL) | 68 (52.7%) |  | 45 (46.9%) |  | 80 (95.2%) |  |
| Total protein, g/L | 77.81 (73.40–79.19) | *0.12* | 77.52 (74.15–83.55) | *0.32* | 76.14 (72.32–81.59) | *0.67* |
| *Albumin, g/L* | 42.10 (38.30–45.40) | ***0.03*** | 42.85 (41.60–45.90) | ***0.01*** | 45.15 (42.05–48.85) | *0.34* |
| *Globulin, g/L* | 36.24 (24.61–47.12) | ***0.01*** | 34.58 (23.54–48.73) | ***<0.001*** | 24.85 (21.61–30.26) | *0.67* |
| *A/G ratio* | 0.91 (0.73–1.62) | ***<0.001*** | 1.21 (0.71–1.69) | ***<0.001*** | 2.36 (1.73–2.81) | *0.07* |
| TBIL, μmol/L | 14.10 (11.15–16.87) | ***0.03*** | 12.97 (10.88–16.75) | *0.47* | 12.23 (10.12–14.11) | ***0.04*** |
| *DBIL, μmol/L* | 4.25 (3.31–5.42) | *0.71* | 4.21 (3.13–5.58) | *0.42* | 4.31 (3.12–5.43) | *0.83* |
| *IDBIL, μmol/L* | 9.54 (6.76–12.01) | *0.55* | 8.94 (6.44–12.02) | *0.89* | 9.01 (7.52–11.13) | *0.43* |
| CRP, mg/L | 0.78 (0.37-1.48) | *0.69* | 1.00 (0.41-2.08) | *0.11* | 0.82 (0.42-1.84) | *0.36* |
| Smoking Status, n (%) |  | *0.43* |  | *0.08* |  | *0.75* |
| *Never* | 40 (31%) |  | 33 (34%) |  | 17 (17%) |  |
| *Former* | 47 (36%) |  | 41 (43%) |  | 32 (39%) |  |
| *Current* | 42 (33%) |  | 22 (23%) |  | 35 (44%) |  |
| Alcohol consumption |  | 0.56 |  | 0.44 |  | 0.79 |
| *None, n (%)* | 69 (53%) |  | 54 (56%) |  | 41 (48%) |  |
| *Mean g/day* | 95.45 (24.56–245.12) |  | 85.25 (31.55–232.10) |  | 69.47 (12.39–287.10) |  |

Data are expressed as the median and inter-quartile range.

BMI: body mass index, calculated as the weight in kilograms divided by the square of height in meters; ALT: alanine aminotransferase; AST: aspartate aminotransferase. TBIL: Total bilirubin; DBIL: Direct bilirubin; IDBIL: Indirect bilirubin; N.A: not applicable.

*P*^a^ refers to the comparison between HCV-monoinfected patients and healthy controls. *P*^b^ refers to the comparison between HCV/HIV-coinfected patients and healthy controls. *P*^c^ refers to the comparison between HCV-monoinfected patients and HCV/HIV-coinfected patients.
